# Treatment of Neurasthenia

**Published:** 1899-02

**Authors:** 


### TREATMENT OF NEURASTHENIA.

Mink in a recent paper (*Medical Bulletin*, Dec.,) discusses this subject with interest; defining it as simply nerve exhaustion, due to impaired nutrition, he proceeds to tell how to overcome it. He says that all of the various symptoms of neurasthenia, be they sensory or motor, psychic or somatic, slowly but surely yield to general systemic and tonic treatment, and disappear, *pari passu*, with the restoration of the nervous system to its normal condition.

In combating insomnia Mink uses drugs only as a last resort. When necessary to use something, however, he finds cannabis indica —10 to 15 minims of the fluid extract—valuable in relieving sleeplessness, mental depression, and the other nerve perturbations that accompany this neurosis. He undertakes to overcome the gastro-intestinal disturbances by attention to diet rather than by drugs, and recommends massage in constipation.

Mink says he has abandoned the various forms of iron and in their place now uses the compounds of arsenic and gold with most satisfactory results. He prefers the liquid bromide of gold and arsenic, known as arsenauro—20 to 30 drops largely diluted with water, after each meal. Finding it, generally speaking, impossible to carry out the Weir-Mitchell rest treatment, he simply advises patients to take as much rest as possible.

---

Abortion.—Stahl, Chicago, (*Canadian Practitioner and Review*) says: Be conservative in the presence of a normally coursed abortion; wait and give nature an opportunity to act. Be radical when dealing with an abnormally coursed abortion; interfere and empty at once.

